# Dashing Growth Curves: a web application for rapid and interactive analysis of microbial growth curves

**DOI:** 10.1186/s12859-024-05692-y

**Published:** 2024-02-12

**Authors:** Michael A. Reiter, Julia A. Vorholt

**Affiliations:** Department of Biology, Institute of Microbiology, ETH Zurich, 8093 Zurich, Switzerland

**Keywords:** Automation, Growth curve, Gompertz, Logistic, Web application, Open source, Microbiology

## Abstract

**Background:**

Recording and analyzing microbial growth is a routine task in the life sciences. Microplate readers that record dozens to hundreds of growth curves simultaneously are increasingly used for this task raising the demand for their rapid and reliable analysis.

**Results:**

Here, we present Dashing Growth Curves, an interactive web application (http://dashing-growth-curves.ethz.ch/) that enables researchers to quickly visualize and analyze growth curves without the requirement for coding knowledge and independent of operating system. Growth curves can be fitted with parametric and non-parametric models or manually. The application extracts maximum growth rates as well as other features such as lag time, length of exponential growth phase and maximum population size among others. Furthermore, Dashing Growth Curves automatically groups replicate samples and generates downloadable summary plots for of all growth parameters.

**Conclusions:**

Dashing Growth Curves is an open-source web application that reduces the time required to analyze microbial growth curves from hours to minutes.

**Supplementary Information:**

The online version contains supplementary material available at 10.1186/s12859-024-05692-y.

## Background

Growth experiments form an integral part of microbial research into single celled organisms. Monitoring microbial abundance over time allows researchers to determine growth parameters under specific conditions, which in turn can be used, for example, to compare different organism, to investigate the effect of media formulations, to quantify the impact of different concentrations of growth inhibiting compounds and the effects of temperature or oxygen availability. Over the past decades, microplate readers that can record many growth curves in parallel have dramatically increased the amount of data an individual researcher can generate. While it used to be a couple dozen samples, it is now routinely hundreds to thousands in one experiment.

Growth curves can be analyzed manually in programs such as Microsoft Excel. However, this is slow and error-prone. To alleviate this problem, we developed Dashing Growth Curves, an easy-to-use open-source web application that streamlines the process of microbial growth analysis. Advancing previous efforts to facilitate growth curve analysis [1–6], the app allows users to interactively explore their data, exclude outliers, fit data using the most common approaches and rapidly analyze the results. To enable a comprehensive understanding of the data, Dashing Growth Curves extracts a complete set of growth parameters including maximum lag time, maximum growth rate, start and end points of exponential growth, number of cell doublings in exponential phase as well as total number of cell doublings between start and end of experiment. With no coding required and in-app user guidance, Dashing Growth Curves enables complete data analysis in minutes instead of hours.

## Implementation

Dashing Growth Curves is written in Python (3.12.0) and uses the Plotly Dash (2.14.0) framework (https://dash.plotly.com/) for the web application logic. Graphs are plotted with Plotly (5.18.0) (https://plot.ly). Data handling is done with pandas (1.5.2) [7]. Numerical calculations and curve fitting are done with Numpy (1.26.3) [8], Scipy (1.11.4) [9] and Uncertainties (3.1.7) [10]. Compute intensive tasks (e.g., curve fitting) are handled on a separate task queue using Celery (5.2.7) (https://docs.celeryq.dev/en/stable/reference/index.html) and Redis (4.3.5) (https://redis.io/). User interface icons are from the Bootstrap icon library (https://getbootstrap.com/). The source code is available on GitHub (https://github.com/mretier/growthdash).

### Growth parameter extraction

Dashing Growth Curves provides the most commonly used methods for determining growth parameter: fitting growth curves to the parametric Logistic and Gompertz growth models, the Easy Linear algorithm and exponential approximation [11]. Noisy data can be smoothed using rolling window averaging. The Logistic and Gompertz growth models were implemented as modified by Zwietering and co-workers (Table 1) [12]. The models contain the maximum growth rate (${\mu }_{max}$), the lag time ($\lambda$) and the maximum population size (${N}_{\infty }$) as parameters. The user can choose between the standard definition of the lag time [12] and an additional definition of lag time referred to herein as “tight”. The former is the timepoint when the tangent at the inflection point of the fitted sigmoidal curve intersects the x-axis [12]. The latter is defined as the timepoint when the slope of the fitted sigmoidal curve increases most (i.e. the smallest zero of the third derivative of the sigmoidal curve) [13], which generally occurs later than the standard definition of the lag phase and is closer to the segment of the sigmoidal curve that can be approximated by a line. Analogously, the end of the logarithmic growth phase occurs when the tangent at the inflection point intersects the line $y=A$ or at the timepoint where the third derivative of the sigmoidal function has its largest zero, respectively. The growth models are fitted to the entire growth curve using non-linear least-squares optimization.

**Table 1:** $N$: Population size; ${N}_{0}$: Population size at time $t=0$; $y={\text{ln}}(\frac{N}{{N}_{0}})$: Logarithmic population size; ${N}_{\infty }$: Maximum population size (${N}_{\infty }={N}_{0} {\text{exp}}(A))$; ${\mu }_{max}$: Maximum growth rate; $\lambda$: Lag time; $t$: Time

Growth model	Equation
Modified logistic	$y=\frac{A}{1+{\text{exp}}\left[\frac{4{\mu }_{\max}}{A}\left(\lambda -t\right)+2\right]}$
Modified Gompertz	$y=A\cdot {\text{exp}}\left\{-{\text{exp}}\left[\frac{{\mu }_{max\cdot e}}{A}\left(\uplambda -{\text{t}}\right)+1\right]\right\}$
Exponential	$N={N}_{0}{\text{exp}}({\mu }_{\max}t)$

The Easy Linear method is a heuristic that fits an exponential growth model (Table 1) to a subsegment of the recorded growth data [4]. First, the algorithms fits a line to all subsegments of a log-transformed growth curve and records the maximum slope. The maximum slope corresponds to the maximum growth rate. The start and end of the exponential phase are defined to be the first and last datapoints of the subsegment exhibiting maximum slope. To improve the automatically generated fits, the size of the subsegments (in datapoints) can be adjusted by the user (e.g., a too small subsegment size will likely overestimate the maximum growth rate and not reflect the actual start and end points of exponential growth).

Growth curves may exhibit irregularities that prevent fully automated analysis and growth parameter extraction using one of the above-described methods. In this case, users can fall back to traditional exponential approximation [14] where the start and end point of exponential growth are defined manually by selecting the subsegment of linear growth in a plot of log-transformed data. As for the Easy Linear method, the start and end points of exponential growth are defined by the first and last datapoints of the selected subsegment.

In addition to the above-described growth parameters, Dashing Growth Curves extracts the number of cell doublings over the whole growth period as well as in exponential growth phase.

### Implementation validation

The implementation of the methods for growth parameter determination was tested using synthetic and real datasets.

Synthetic Logistic and Gompertz growth curves were generated using random growth parameters with and without random noise added ($n=100$ individual growth curves). All growth parameters were sampled randomly from uniform distributions with biologically relevant ranges (${N}_{0}\sim {\text{U}}(0, 0.5)$, $A\sim {\text{U}}\left(0.5, 4\right), {\mu }_{max}\sim {\text{U}}(0, 2)$, $\lambda \sim {\text{U}}(0, 100)$. Noise was sampled from a Normal distribution and added to the previously generated growth curves. Fitting the synthetic data with their respective Logistic or Gompertz growth models reliably recovered the growth parameters (median relative error for data without noise = 0.000, median relative error for data with noise ranged from 0.003 to 0.071, Additional file 1: Data 1).

The real dataset came from *Escherichia coli* growth curves recorded in our lab. Fitting the real dataset with the Logistic or Gompertz growth models resulted in good fits in cases where growth was well approximated by these models (Additional file 2: Fig. S1A and B). Independent of the overall growth curve shape, the Easy Linear algorithm detected the exponential growth phase well (R^2^ > 0.98) in the test datasets but was dependent on the user setting the window size appropriately (Additional file 2: Fig. S1C).

All scripts and datasets used for evaluation are available through the GitHub repository (https://github.com/mretier/growthdash/tree/main/implementation_test).

### Data privacy and local installation

Dashing Growth Curves does not store user data nor uploaded data beyond the time it is in active use in the web application. Data uploads are not logged. Cookies are not set. Additionally, Dashing Growth Curves can be installed locally within a few minutes following a short manual (see the readme in the GitHub repository). The local installation is identical (except for the backend implementation) to the hosted web version.

## Results and discussion

Dashing Growth Curves can be accessed via the internet using any common web browser (http://dashing-growth-curves.ethz.ch/). However, the application has been optimized for best display in Google Chrome.

**Fig. 1:**
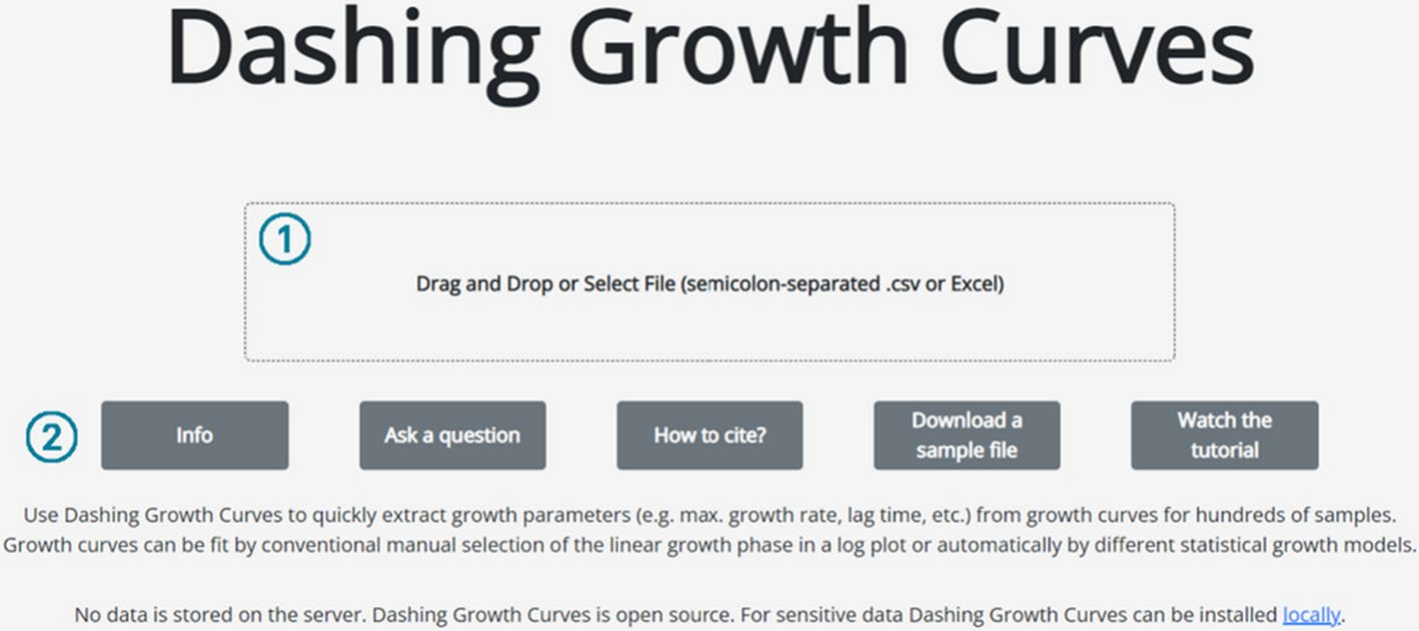
Dashing Growth Curves landing page. ① Drag and drop the data file into the box to upload it to the application or click inside the box to open a dialog box to select it. ② Different resources. “Info” forwards to the GitHub repository (https://github.com/mretier/growthdash) which contains all information about Dashing Growth Curves. “Ask a question” forwards to the associated GitHub issues page where bugs can be reported or questions about the application can be raised (https://github.com/mretier/growthdash/issues). “How to cite” forwards to the most recent associated publication of Dashing Growth Curves. “Download a sample file” downloads an Excel file with correctly formatted growth curve data. “Watch the tutorial” forwards to a video tutorial explaining Dashing Growth Curves (https://www.youtube.com/watch?v=lhvgZyPlHzA)

**Fig. 2:**
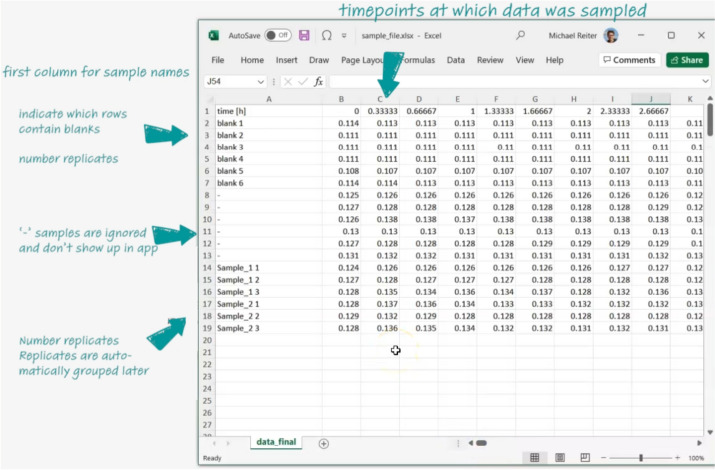
Data structure of a set of individual growth curves

On the Dashing Growth Curves landing page users can upload their data and are offered different resources (Fig. 1). For Dashing Growth Curves to be able to parse growth data, it needs to be in a simple table format and contain timestamps and sample names (Fig. 2). After data upload, it is embedded in a graphical user interface.

**Fig. 3:**
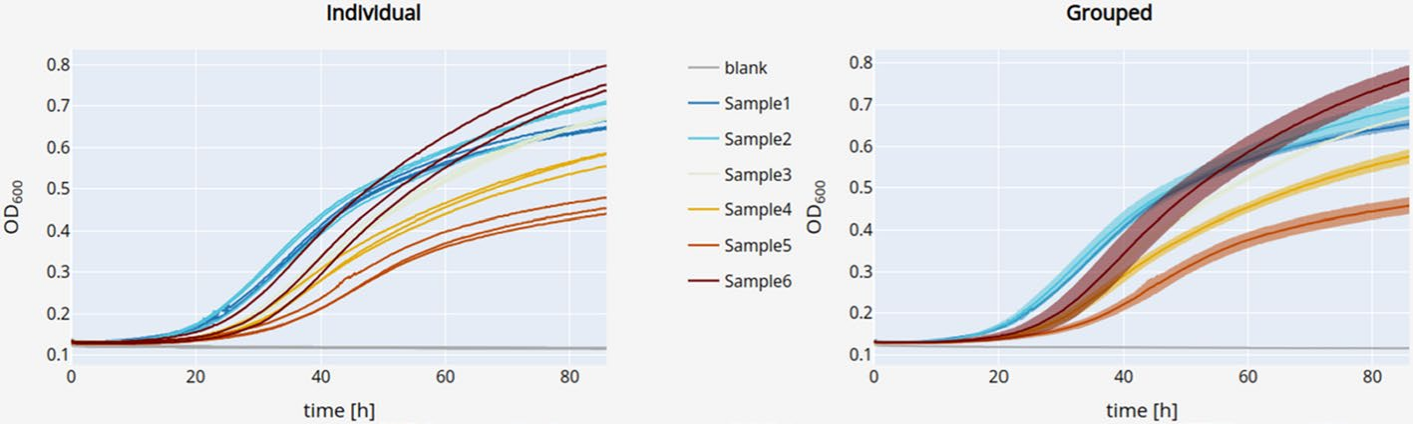
Data exploration view. Uploaded data is displayed as individual traces of each replicate (left plot) or as traces representing the mean of all replicates belonging to the same sample (right plot). Error bands around the mean traces depict the standard deviation. Traces of replicates belonging to the same sample are colored identically. Data can be explored interactively through panning, zooming and hiding individual traces

**Fig. 4:**
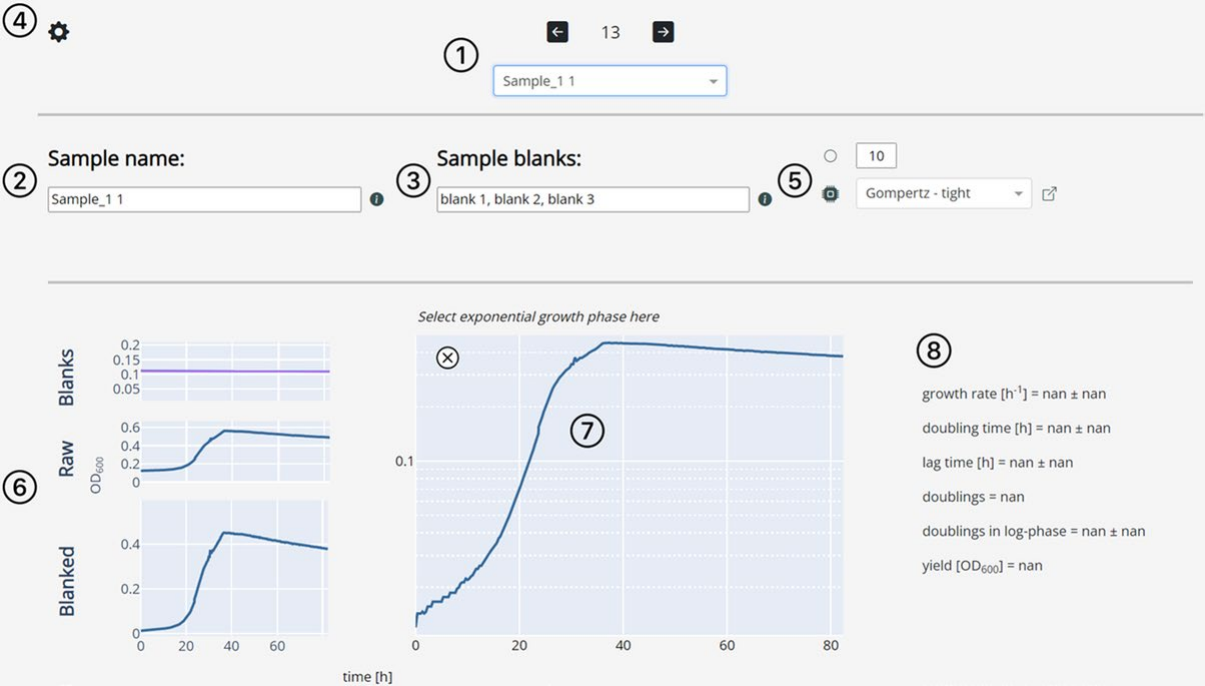
Dashing Growth Curves sample view. ① Position of the currently selected sample and its name. The dropdown menu or the arrow buttons can be used to change the currently displayed sample. If there are 6, 12, 24, 96 or 384 samples, a sample position will be associated with each sample (e.g. the 13th row in a data file of 96 samples will be position B2) if there are a number of samples that are not associated with a standard microtiter plate size, each will be associated with a number. ② The sample name can be changed here. ③ The field displays the blanks associated with the current sample. The associated blanks can be changed by providing a list of sample names that are to be used as blanks. By default, Dashing Growth Curves takes the first three samples in a data file as blanks. The default blanks for all samples can be changed in ④. ⑤ Circle button: data can be smoothed using a rolling average algorithm (the default window is 10 data points). CPU button: Fit all growth curves to a chosen parametric growth model or with the Easy Linear algorithm (the option can be selected through the dropdown menu). The button to the right of the dropdown menu leads to the documentation of the different automatic growth parameter extraction approaches. ⑥ Graphs of the associated blanks and their mean (here all three blanks are very similar and fall onto one line), the raw trace of the currently selected sample and the blanked trace. ⑦ The blanked trace plotted on a logarithmic y-axis. Pressing the 'x' button excludes the sample from the summary view (Fig. 4). ⑧ Growth parameters of the currently selected sample. If the logarithmic growth phase has not been determined manually or the sample has not been fitted to a parametric growth model yet, the values are ‘nan’ (not a number)

**Fig. 5:**
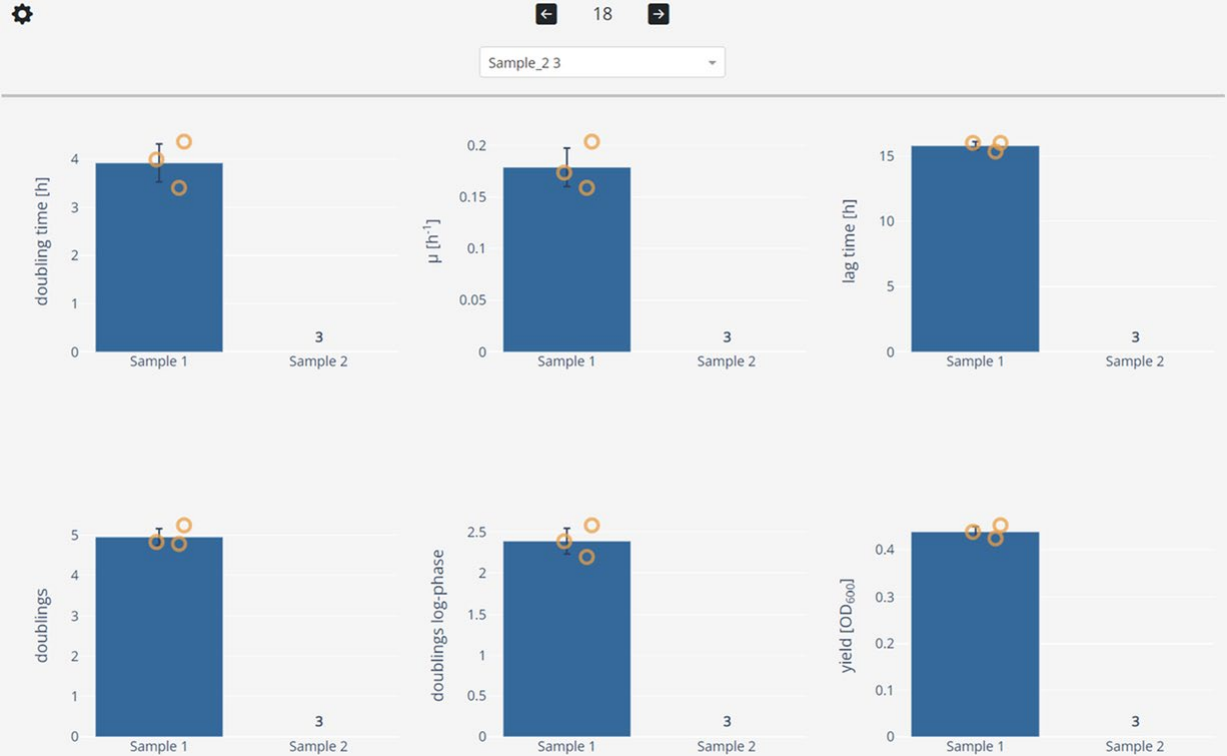
Dashing Growth Curves summary view. Sample replicates are automatically grouped. For each group and every growth statistic a bar chart is plotted on top of which the individual datapoints are overlayed. Clicking on a datapoint selects it and displays its growth curve in the sample view (e.g., to doublecheck outliers). Individual growth curves can be excluded (e.g., for technical reasons). Here, for ‘Sample 2’ all three replicates were excluded. The number of excluded samples is tracked by a counter above the sample name

Three views of the data are available. The “overview view” displays the whole dataset and enables rapid data exploration (Fig. 3). The “sample view” shows the growth curve of a sample and its associated growth parameters and blanks (Fig. 4). The “summary view” plots all computed growth parameters and groups replicate samples (Fig. 5). Dashing Growth Curves extracts the doubling time, the maximum growth rate, the lag time, the number of doublings from lowest to highest measured population size, the doublings in logarithmic growth phase and the yield (i.e., the maximum population size) for every sample. If no logarithmic growth phase is observed, the associated parameters are left undetermined. By default, the population size is indicated by optical density measurements at 600 nm (OD_600_), but this can be changed in the settings menu (e.g., to colony forming units, Fig. 4④).

**Fig. 6:**
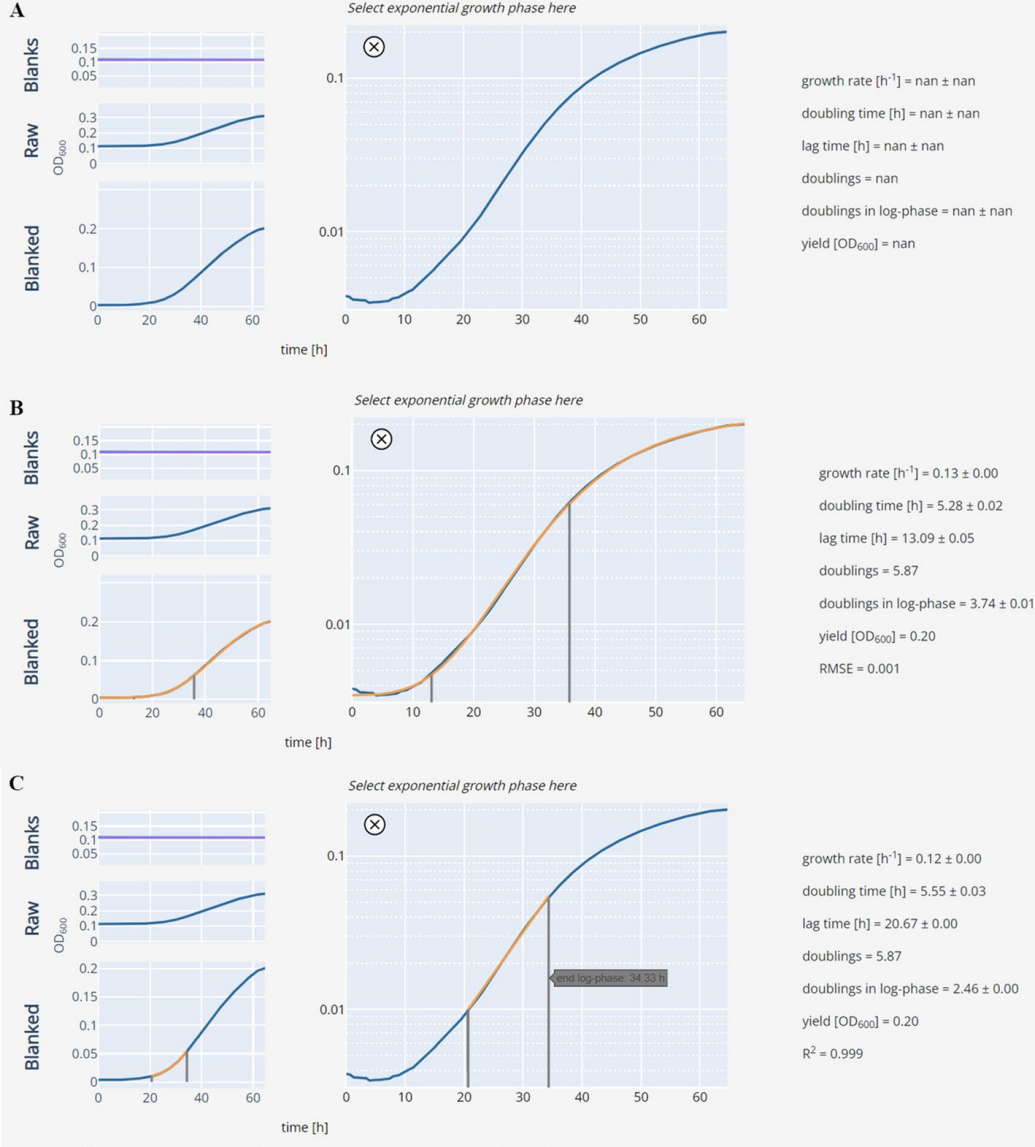
Fitted growth curve examples. **A** Raw, unfitted growth curve. **B** Growth curve fitted with a Gompertz model (see “Fitting growth curves” section). The fit is overlayed in orange. The grey lines indicate the beginning and end of the logarithmic growth phase. **C** Growth curve fitted manually by selecting the segment in which the observed logarithmic growth over time is linear

To determine growth parameters, the most commonly used approaches are available [11]. Data can be fitted automatically with parametric models (Fig. 6A, B and section “Fitting growth curves”) or the Easy Linear algorithm. Alternatively, each growth curve can be analyzed individually by manually selecting the segment of the growth curve that exhibits a linear increase over time in a logarithmic plot and fitting a line (Fig. 5C). Manual selection, while being slower and not taking into account the whole growth curve, provides greater flexibility for growth patterns that are not captured by the Logistic or Gompertz growth models (not all microbial growth follows these models and limitations include, e.g., diauxic growth or presence of death phase) nor the Easy Linear algorithm (e.g. noisy data).

To facilitate further analysis and data presentation, all plots can be downloaded as editable vector graphics (.svg files, Fig. 7①) or interactive HTML plots (Fig. 7②). Furthermore, a summary file that contains all data can also be downloaded for further analysis or custom plotting (Fig. 7③).

**Fig. 7:**
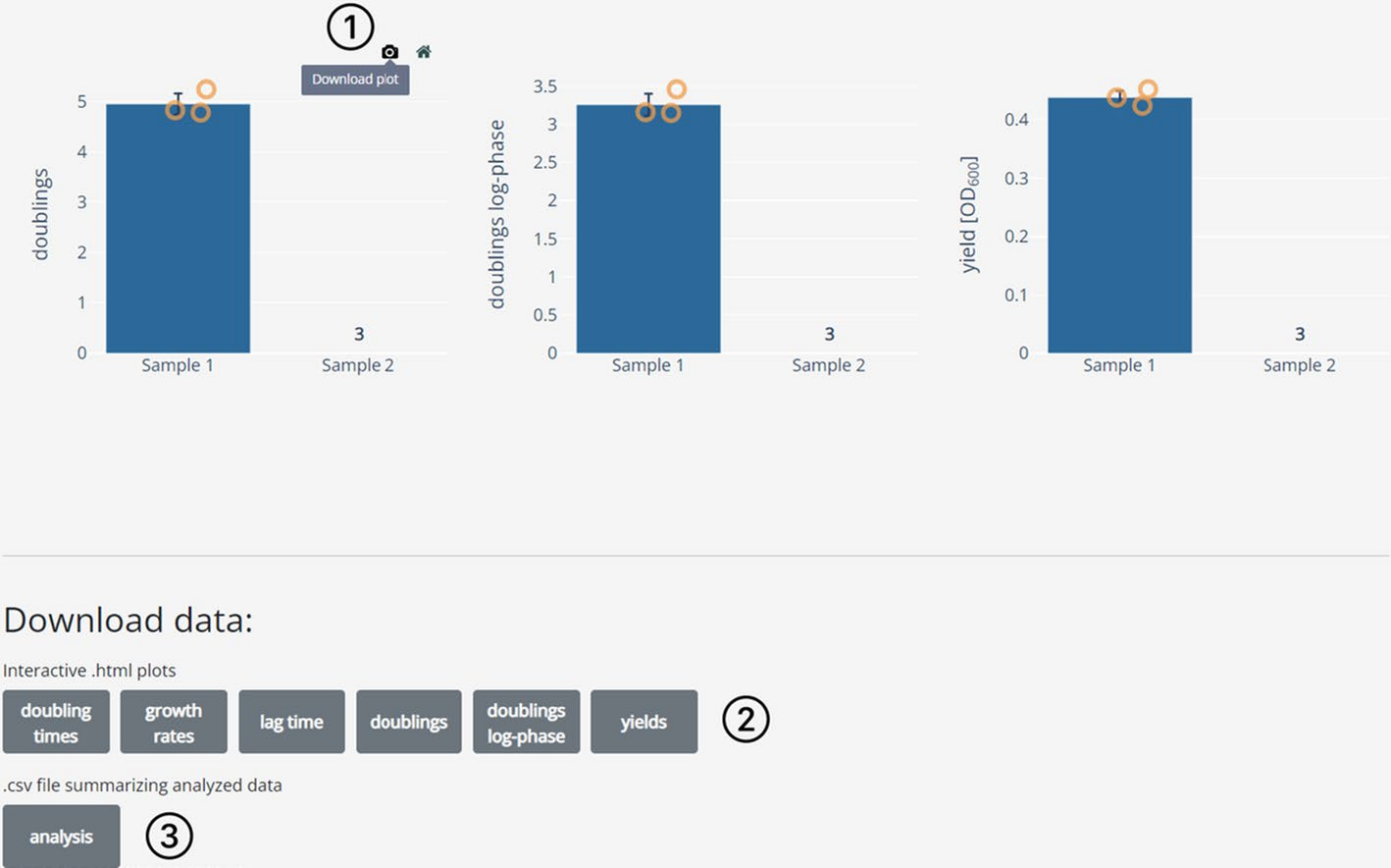
Dashing Growth Curves data download options. ① Hovering on the top right corner of every plot shows an option to download the respective graph as a vector graphic. ② The same graphs can also be downloaded as interactive HTML plots. ③ The extracted growth parameters for all samples can be downloaded as a comma separated values (.csv) file

Lastly, Dashing Growth Curves can handle up to thousands of growth curves each with hundreds of time-resolved measurements (we tested datasets with up to half a million data points, i.e., the number of samples multiplied by the number of timepoints recorded per sample).

## Conclusions

Here, we introduce Dashing Growth Curves, an open-source web application to support researchers in analyzing growth curves quickly and reliably. Dashing Growth Curves requires no programming skills and is operating system independent. Lastly, it gives users the flexibility to fit their data with different growth models or to use the traditional approach of manual selection of the logarithmic growth phase.

## Availability and requirements

Project name: Dashing Growth Curves

Project home page: https://github.com/mretier/growthdash

Operating system: operating system independent

Programming language: Python 3.12

License: GNU General Public License v3.0

Any restrictions to use by non-academics: None

Other requirements: None

### Supplementary Information

**Additional file 1.** Quality control fitting errors.

**Additional file 2. Supplementary Figure 1.** Growth curve fitting validation. **A** Logistic growth model fits. **B** Gompertz growth model fits. **C** Easy Linear growth model fits. In all cases three growth curves were randomly selected out of a dataset of 18 real recorded growth curves. The dataset is available in the GitHub repository (https://github.com/mretier/growthdash/tree/main/implementation_test).

## Data Availability

All data and code are available through the associated GitHub repository (https://github.com/mretier/growthdash).
