# Supplementary figures and images for: Dashing Growth Curves: a web application for rapid and interactive analysis of microbial growth curves
Source: BMC Bioinformatics. 2024 Feb 12;25:67. doi: 10.1186/s12859-024-05692-y (PMC10863085; doi:10.1186/s12859-024-05692-y)

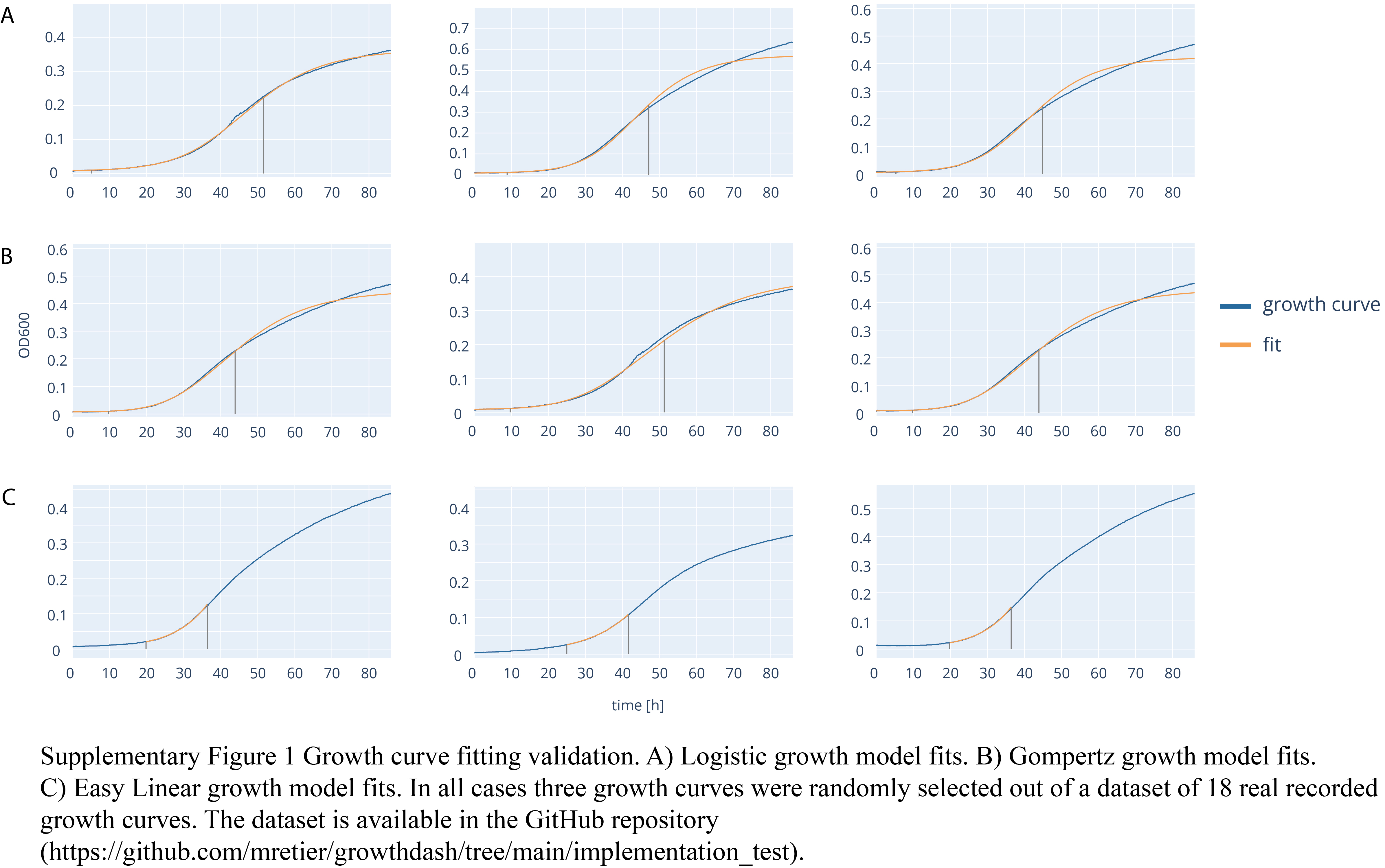

Supplement: Supplementary file 2 — Additional file 2. Supplementary Figure 1. Growth curve fitting validation. A Logistic growth model fits. B Gompertz growth model fits. C Easy Linear growth model fits. In all cases three growth curves were randomly selected out of a dataset of 18 real recorded growth curves. The dataset is available in the GitHub repository (https://github.com/mretier/growthdash/tree/main/implementation_test). [file 12859_2024_5692_MOESM2_ESM.jpg]
